# Sweet Syndrome in a Patient with Chronic Lymphocytic Leukemia/Small Lymphocytic Lymphoma: Curious Lymphocyte/Neutrophil Fluctuations

**DOI:** 10.4274/Tjh.2012.0055

**Published:** 2013-12-05

**Authors:** Çiğdem Usul Afşar, Semra Paydaş, Meral Günaldı, Berna Bozkurt Duman, Vehbi Erçolak, Suzan Zorludemir, Arbil Açıkalın

**Affiliations:** 1 Çukurova University Medical Faculty, Department of Medical Oncology, Adana, Turkey; 2 Çukurova University Medical Faculty, Department of Pathology, Adana, Turkey

**Keywords:** Chronic lymphocytic leukemia, fever, malignancy, Neutrophilic leucocytosis, Steroid, Sweet syndrome

## Abstract

Sweet syndrome, also referred to as acute febrile neutrophilic dermatosis, is characterized by tender, red inflammatory nodules or papules that occur in association with infection, malignancy, connective tissue disease, or following exposure to certain drugs. Here, we present Sweet syndrome in a case with small lymphocytic lymphoma/chronic lymphocytic leukemia (SLL/CLL) which is a relatively rare co-occurrence.

**Conflict of interest:**None declared.

## Abstract (tr)

Akut febril nötrofilik dermatoz da denilen Sweet sendromu; enfeksiyon, malignite, konnektif doku hastalıkları veya bazı ilaçlara maruz kalma sonucu ortaya çıkan; kırmızı, hassas inflamatuvar nodüller veya papüllerle karakterizedir. Burada oldukça nadir bir birliktelik olan küçük lenfositik lenfoma/kronik lenfositik lösemili (SLL/CLL) bir vakada gelişen Sweet sendromunu sunduk.

## INTRODUCTION

In 1964 Robert D. Sweet described a new entity called “acute febrile neutrophilic dermatosis” and he characterized the features of the disease. Also known as Sweet syndrome (SS), it is characterized by a sudden onset of symptoms including fever, neutrophilia, and characteristic skin lesions [1]. However, this disease is not limited to the skin; other organs may be involved. There are various types, including idiopathic, para-inflammatory, drug-induced, pregnancy-related, and paraneoplastic disease [2,3,4,5]. Paraneoplastic disease may be associated with hematological disorders, mainly myeloproliferative diseases [6]. About 85% of the cases have hematopoietic neoplasias, and 15% have solid tumors [3]. Small lymphocytic lymphoma/chronic lymphocytic leukemia (SLL/CLL) is a relatively rare entity accompanying SS and a few cases have been reported so far [7,8]. 

## CASE REPORT

**Figure 1:**
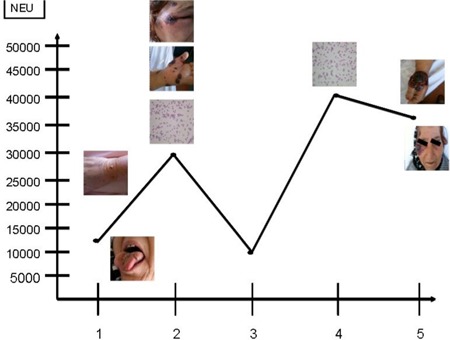
Mouth, skin lesions and neutrophilic fluctuations in the patient

**Figure 2:**
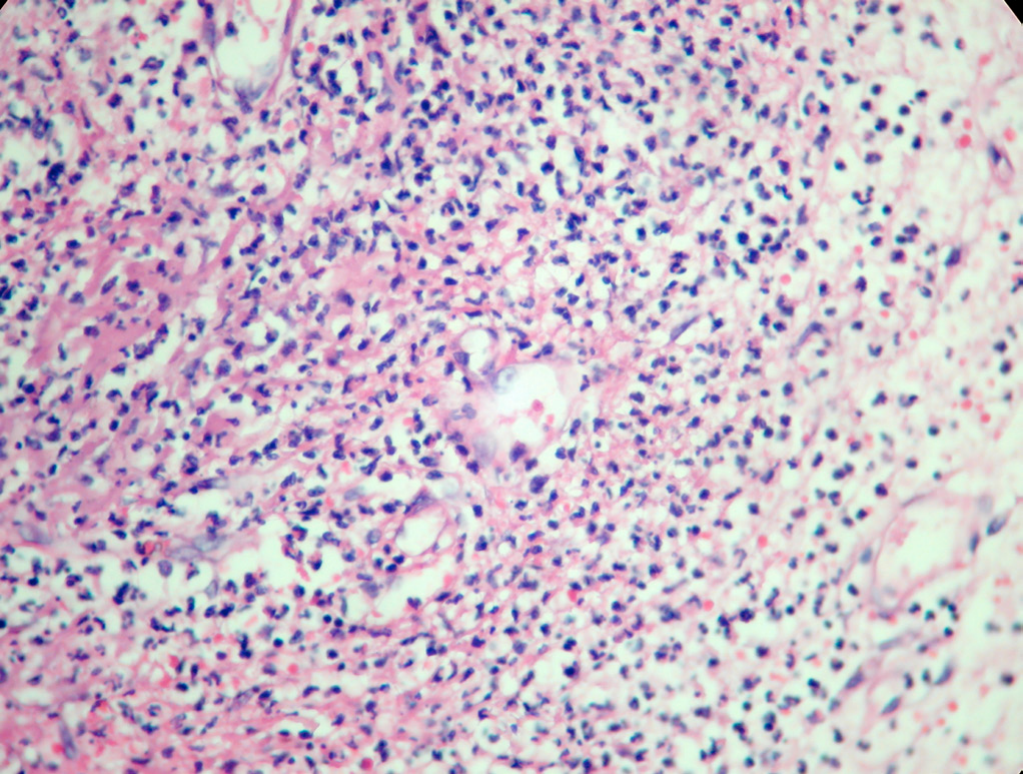
Dense perivascular infiltration of the dermis composed largely by neutrophils

**Figure 3:**
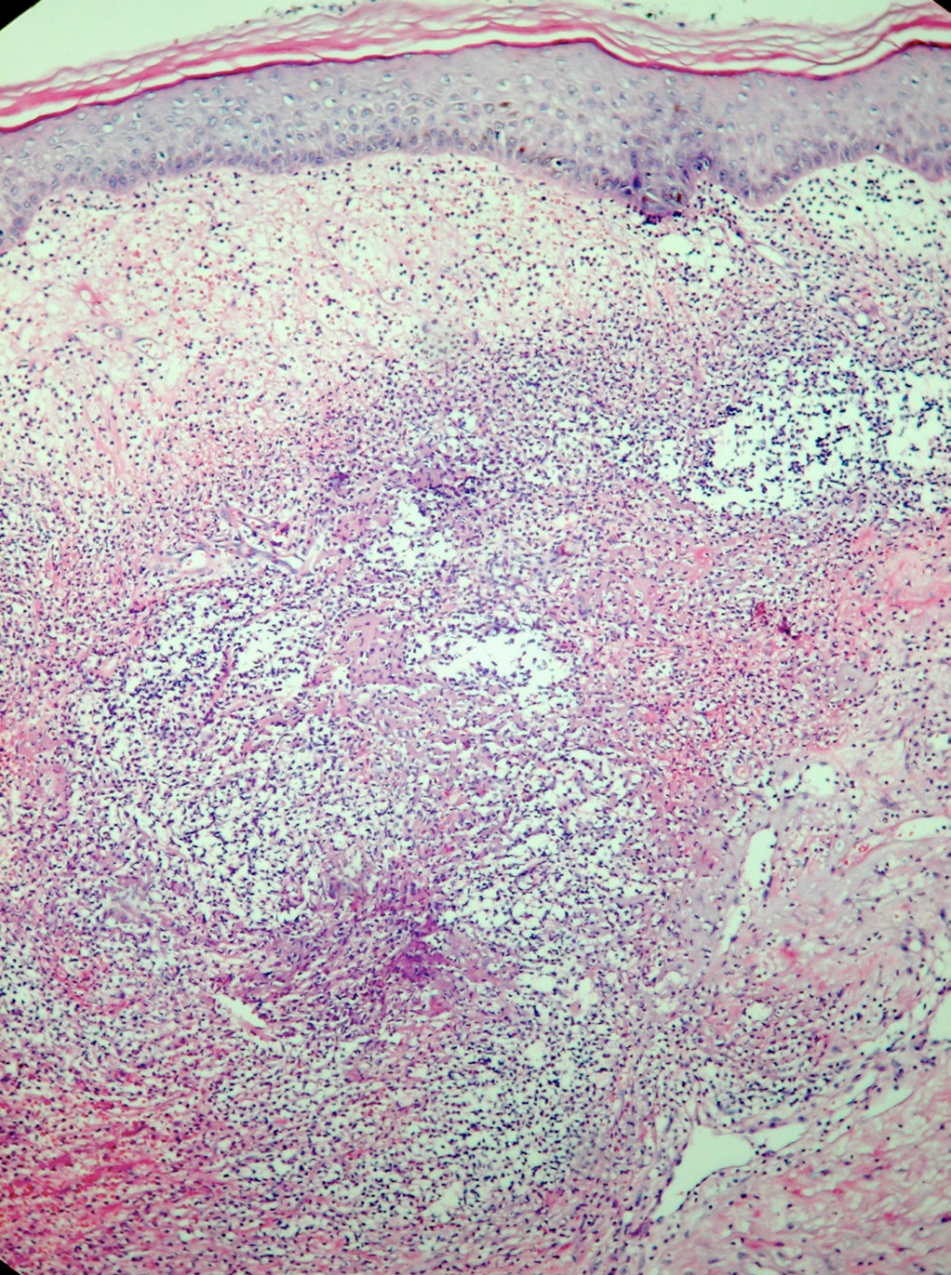
Neutrophilic infiltration, vasodilatation, endothelial swelling and erythrocyte extravasation

A 58-year-old woman was admitted to our hospital with fever, nausea, abdominal pain, and oral ulcer. She had a 10-year history of SLL/CLL. Initially, she had been treated with cyclophosphamide, vincristine, and prednisolone (CVP). Relapse developed 5 years later and she was then treated with a regimen of rituximab, fludarabine, and cyclophosphamide (R-FC). In 2006, when she was in remission for CLL/SLL, a lung mass was detected and a hemangioma was diagnosed on biopsy. At that time there was no evidence of SS and she had no symptoms due to disease until she presented with fever, large aphthous lesions on the tongue, and hepatosplenomegaly. Informed consent was obtained. The patient was not using any drugs or herbal products. Mouth and skin lesions, fever, and neutrophilic fluctuations in the patient clinically suggested SS (Figure 1, Table 1), and a biopsy was taken. Initial complete blood cell as follows were: white blood cells, 13.9 × 10^9^/L; neutrophils, 7.46 × 10^9^/L, hemoglobin, 9.6 g/dL, and platelets, 16.9 × 10^9^/L. Bone marrow aspiration showed lymphocytosis. One week later, neutrophilic leukocytosis occurred, which was confirmed by peripheral smear, and at that time, the skin lesions worsened. The pathological examination revealed inflammatory infiltrate (neutrophils) located in the dermis, epidermis, and adipose tissue. Microscopically, there was dense perivascular infiltration of the dermis composed largely by neutrophils (Figure 2), vasodilatation, endothelial swelling, and erythrocyte extravasation (Figure 3), with prominent edema of the upper dermis. Prednisolone was initiated; the fever disappeared within 12 hours and the mucosal lesions improved dramatically. However, during follow-up, purpuric, hemorrhagic, and necrotic painful skin lesions had developed, and fever had recurred at the end of the fourth week. Clinically, these lesions also suggested SS, and a biopsy was obtained. Pulse steroid (1 g daily) was given, but the response was not as good as it was earlier; skin lesions showed progression, and clinical symptoms suggested myositis. Computed tomography (CT) revealed consolidations in the lungs. Due to the difficulty in excluding an opportunistic infection and underlying CLL/SLL, piperacillin/tazobactam (4 × 4.5 g), acyclovir Introduction 

## DISCUSSION

Sweet syndrome is an interesting entity and most commonly accompanies myeloid neoplasias. Sweet syndrome accompanying CLL/SLL is rare, and so far, few cases have been reported. The pathogenesis of this entity is not clear yet, but hematopoietic growth factors seem to play important role in the mechanism of disease; thus increased use of these cytokines may potentially increase the incidence of this entity. Among these growth factors, granulocyte colony-stimulating factor (G-CSF), both endogenous and exogenous, is the most important factor in the development of the signs and symptoms of the disease [9]. The clinical importance of SS with respect to hematologic malignancies is that it can be the very early sign of an indolent or recurrent malignancy. 

Mucosal involvement of the mouth, appearing as oral or vaginal ulcers, is uncommon in patients with classical SS. Mucosal SS suggests an underlying malignancy, an important point in daily practice. In our patient, oral lesion was the first sign of SS, and skin lesions developed later. At the beginning, dramatic response to steroids was observed, with fever and oral lesion disappearing within 12 hours. Another very interesting point was that there was neutrophilia in the peripheral blood while there was lymphocyte infiltration in the bone marrow. After a short remission with steroids, skin lesions developed. Concomitant muscle pains suggesting myositis and CT changes in the lungs were detected, along with more severe neutrophilia, as high as 50 × 109/L. There was no evidence of an autoimmune process. At that time, a high dose of corticosteroid was given; a minimal response was observed but skin and muscle problems increased. Fever recurred despite the use of broad-spectrum antibiotics in addition to the pulse steroid, and her condition deteriorated. At the end, a complicated clinical picture suggesting thrombotic thrombocytopenic purpura developed and she died.

Systemic corticosteroids are the therapeutic gold standard for SS. After initiation of treatment with systemic corticosteroids, there is a prompt response consisting of dramatic improvement of both the dermatosis-related symptoms and the skin lesions. There was a good response at first in our patient, but she relapsed quickly after discontinuation of the steroids.

In conclusion, SS is rare in patients with SLL/CLL. It should be kept in mind that this syndrome can accompany malignancies and cannot be managed easily as an idiopathic form. This report emphasizes the association of SS with SLL/CLL, and very high neutrophil levels in spite of lymphocyte infiltration in bone marrow is the other interesting and important point. 

## CONFLICT OF INTEREST STATEMENT

The authors of this paper have no conflicts of interest, including specific financial interests, relationships, and/ or affiliations relevant to the subject matter or materials included.

## Figures and Tables

**Table 1:**
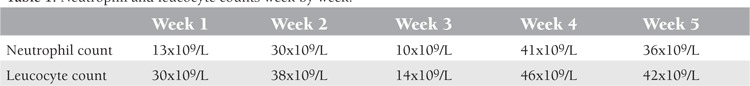
Neutrophil and leucocyte counts week by week
